# Prognostic Value of Non-Invasive Global Myocardial Work in Asymptomatic Aortic Stenosis

**DOI:** 10.3390/jcm11061555

**Published:** 2022-03-11

**Authors:** Federica Ilardi, Adriana Postolache, Raluca Dulgheru, Mai-Linh Nguyen Trung, Nils de Marneffe, Tadafumi Sugimoto, Yun Yun Go, Cécile Oury, Giovanni Esposito, Patrizio Lancellotti

**Affiliations:** 1 Department of Cardiology and Radiology, GIGA Cardiovascular Sciences, CHU Sart Tilman, Liege University Hospital, 4000 Liege, Belgium; federica.ilardi@unina.it (F.I.); adriana.postolache@gmail.com (A.P.); eralucadulgheru@yahoo.com (R.D.); mlnguyentrung@gmail.com (M.-L.N.T.); ndemarneffe@chuliege.be (N.d.M.); t_sugimoto_japan@hotmail.com (T.S.); go.yun.yun@singhealth.com.sg (Y.Y.G.); cecile.oury@ulg.ac.be (C.O.); 2 Department of Advanced Biomedical Sciences, Federico II University Hospital, Via S. Pansini, 5, 80131 Napoli, Italy; espogiov@unina.it; 3 Clinical Laboratory, Mie University Hospital, Tsu 514-8507, Japan; 4 Department of Cardiology, National Heart Research Institute Singapore, National Heart Centre Singapore, Singapore 169609, Singapore; 5 Gruppo Villa Maria Care and Research, Anthea Hospital, 70124 Bari, Italy

**Keywords:** aortic stenosis, asymptomatic, myocardial work, cardiac damage, staging, prognosis

## Abstract

This study aimed to evaluate the modification of non-invasive myocardial work (MW) indices related to aortic stenosis (AS) stages of cardiac damage and their prognostic value. The echocardiographic and outcome data of 170 patients, with asymptomatic moderate-to-severe AS and left ventricular ejection fraction (LVEF) ≥ 50%, and 50 age- and sex-comparable healthy controls were analysed. Primary endpoints were the occurrence of all-cause and cardiovascular death. Increased values of the global work index (GWI), global constructive work (GCW), and global wasted work (GWW) were observed in AS patients compared to controls (GWI: 2528 ± 521 vs. 2005 ± 302 mmHg%, GCW: 2948 ± 598 vs. 2360 ± 353 mmHg%, *p* < 0.001; GWW: 139 ± 90 vs. 90 ± 49 mmHg%, *p* = 0.005), with no changes in the global work efficiency. When patients were stratified according to the stages of cardiac damage, the GWI showed lower values in Stage 3–4 as compared to Stage 0 and Stage 2 (*p* = 0.024). During a mean follow-up of 30 months, 27 patients died. In multivariable Cox-regression analysis, adjusted for confounders, GWI (HR: 0.998, CI: 0.997–1.000; *p* = 0.034) and GCW (HR:0.998, CI: 0.997–0.999; *p* = 0.003) were significantly associated with excess mortality. When used as categorical variables, a GWI ≤ 1951 mmHg% and a GCW ≤ 2475 mmHg% accurately predicted all-cause and cardiovascular death at 4-year follow-up. In conclusion, in asymptomatic patients with moderate-to-severe AS, reduced values of GWI and GCW are associated with increased mortality. Therefore, the evaluation of MW indices may allow for a better identification of asymptomatic patients with moderate to severe AS and preserved LVEF whom are at increased risk of worse prognosis during follow-up.

## 1. Introduction

According to the current guidelines, aortic valve replacement (AVR) in patients with severe aortic stenosis (AS) and preserved left ventricular ejection fraction (LVEF) is driven by the occurrence of symptoms [1]. Conversely, in asymptomatic patients, intervention should be considered in the presence of very severe AS, given that some haemodynamic parameters (peak aortic jet velocity ≥ 5 m/s, rapid disease progression, severe pulmonary hypertension) have been associated with increased mortality [1,2]. Regardless of the symptoms, patients with moderate AS have shown a comparable risk of death to patients with severe AS [3]. Moreover, when asymptomatic moderate-to-severe AS patients were stratified by stage of cardiac injury, an increased mortality rate was observed in advanced stages [4]. This would suggest that a more comprehensive risk assessment should be performed by considering not only Doppler imaging data but also structural and haemodynamic cardiac changes.

In recent years, myocardial work (MW) has become an alternative tool for the assessment of myocardial function [5]. This new parameter, estimating LV function in a less load-dependent manner through the evaluation of strain in relation to the dynamic non-invasive LV pressure, could be particularly useful in pathological conditions, such as AS, characterized by an increased afterload. To date, the role of MW in identifying cardiac dysfunction in AS and predicting prognosis has not been investigated. The aim of the present study was to evaluate the modification of MW indices related to the stages of AS and their prognostic value.

## 2. Materials and Methods

### 2.1. Patient Population

We retrospectively analysed echocardiographic data of 227 patients with moderate-to-severe AS and preserved LVEF, who were prospectively followed-up with in our heart valve clinic. All patients met the following criteria: age > 18 years old, aortic valve area ≤ 1.5 cm^2^, LVEF ≥ 50% as calculated by 2D echocardiography, no more than moderate associated cardiac valve lesions not AS-related, absence of symptoms and uncontrolled hypertension at baseline, and good image quality. Patients with known coronary artery disease (defined as the presence of atherosclerotic plaque accumulation in the epicardial arteries, whether obstructive or non-obstructive) or previous percutaneous coronary intervention were included in the study. Exclusion criteria were left bundle branch block and previous heart surgery, including coronary artery bypass graft. Patients’ symptomatic status were carefully assessed by experienced physicians from direct patient interview and physical examination. In patients with equivocal history or symptoms, exercise testing was performed to detect truly asymptomatic patients. In total, 18 patients were excluded for rhythms disturbance, 39 for suboptimal quality of speckle-tracking image analysis. The final study population consisted of 170 patients. The control group included 50 patients matched for age and sex (data derived from the NORRE study) [6].

### 2.2. Echocardiographic Measurements

Transthoracic echocardiograms were performed using a Vivid ultrasound (7, E9 or E95) System (GE Healthcare, Horten, Norway) and stored on a dedicate workstation for off-line analysis (EchoPAC, GE Healthcare, Version 202). Conventional echocardiographic measurements were performed in accordance with the guidelines [7]. Valvulo-arterial impedance (Zva) was calculated as the sum of systolic blood pressure and mean transaortic gradient, divided by indexed LV stroke volume [8]. Myocardial mechano-energetic efficiency was calculated as the ratio of stroke volume and heart rate, where stroke volume was calculated using the z-derived method to estimate LV volumes [9]. Global longitudinal strain (GLS) analysis was measured as previously described [10]. Right ventricular (RV) LS was calculated as the average of regional strain from RV free wall segments and interventricular septum.

### 2.3. Myocardial Work Analysis

Quantification of MW was performed using commercially available software package (Echopac Version 202, GE Healthcare). As proposed by Russel et al. [11] and previously described by our group [6], MW was estimated as the area of the pressure–strain loops (PSL), which were derived by a combination of speckle tracking-derived LV strain data and non-invasive LV pressure curves. In the control group, peak systolic LV pressure was assumed to be equal to the brachial systolic blood pressure measured with a cuff manometer. In patients with AS, LV systolic pressure is supposed to be increased due to an increase in transvalvular pressure gradient [8]. As previously validated by Jain et al. [12] and Fortuni et al. [13], peak systolic LV pressure was estimated as the sum of systolic blood pressure measured with a cuff manometer and mean transaortic pressure gradient measured at echocardiography. Then, the patient-specific, non-invasive pressure curve was aligned to the valvular event times, which were set by pulse-wave Doppler recordings at mitral valve and aortic valve level and then confirmed by two-dimensional echocardiographic evaluation of the apical long-axis view [5]. Total work within the area of the PSL provided the measure of global work index (GWI). Moreover, additional indices of MW were obtained as follows: global constructive work (GCW, work performed during shortening in systole adding negative work during lengthening in isovolumetric relaxation); global wasted work (GWW, negative work performed during lengthening in systole adding work performed during shortening in isovolumetric relaxation); global work efficiency (GWE, constructive work divided by the sum of constructive and wasted work) [5,6].

### 2.4. Cardiac Damage Staging Classification

On the basis of the cardiac damage staging scheme recently proposed by Tastet et al. [4], patients were classified as follows: stage 0, no cardiac damage; stage 1, LV damage as defined by the presence of LV hypertrophy (LV mass index >95 g/m^2^ in women and >115 g/m^2^ in men) and/or LV diastolic dysfunction ≥ grade II, and/or LV subclinical systolic dysfunction (LVEF <60%, GLS ≤ −15%); stage 2, left atrial (LA) and/or mitral valve damage as defined by LA volume > 34 mL/m^2^ and/or more than mild mitral regurgitation and/or the presence of atrial fibrillation; stage 3, pulmonary hypertension (systolic pulmonary artery pressure at rest ≥ 60 mmHg) or tricuspid valve damage (more than mild tricuspid regurgitation); stage 4, RV damage based on parameters of longitudinal function (value of TAPSE < 17 mm and s’ < 9.5 cm/sec) or subclinical heart failure with moderate to severe low-flow (stroke volume index < 30 mL/m^2^). As the RV strain was not available for all patients, this parameter was not used for defining RV dysfunction. Patients were hierarchically classified in a given stage (worst stage) if at least 1 of the proposed criteria was met. Given that there was a small number of patients in Stage 3 and that previous data [4] reported similar outcomes for patients in Stages 3 and 4, these 2 stages were merged in a single group (Stages 3–4).

### 2.5. Clinical Follow-Up and Endpoints

Patients were routinely followed-up and managed according to available guidelines, and clinical information was obtained from direct patient interview telephone calls with physicians, patients, or next of kin, or review of autopsy records and death certificates. Primary endpoints were the occurrence of all-cause and cardiovascular-related mortality.

### 2.6. Statistical Analysis

Data are reported as mean ± standard deviation for continuous variables or counts with percentages of individuals for categorical variables. Group comparisons were performed using two-sample *t*-test and Chi-square test for continuous and categorical variables. One-way analysis of variance (ANOVA) test and Bonferroni post hoc test was used to compare staging groups. Multivariable Cox proportional hazards model adjusted for age, sex, body mass index, BNP (log_e_), LV mass index, indexed LA volume, GLS, GWI, GCW, and AVR was used to determine the independent association with mortality. The selection of the variables for the multivariable analysis was based on their significant association (*p*-value < 0.1) with mortality in univariable analysis. Two models were generated to avoid collinearity between MW parameters. Receiver Operator Characteristics (ROC) curves were generated, and the Youden’s J statistic was used to estimate the best cut-off value that predicted death. The Kaplan–Meier method was used for cumulative survival analysis with the log-rank test for assessing statistical differences between curves. Statistical analyses were performed using IBM-SPSS, version 23 (SPSS Inc., Chicago, IL, USA). A *p*-value < 0.05 was considered significant. Reproducibility analyses were previously published by our group [7].

## 3. Results

### 3.1. Patients’ Characteristics

The clinical and echocardiographic characteristics of the study group and the controls are presented in Table 1. Compared to the controls, patients with AS had higher systolic and diastolic arterial blood pressure (*p* < 0.05) and increased LV wall thickness (*p* < 0.005), LV mass index (*p* < 0.001) and end-systolic volume (*p* = 0.025). A more pronounced diastolic dysfunction, an increased systolic pulmonary artery pressure (*p* < 0.001) and an impaired RV function (*p* < 0.05) were also observed in AS patients. Despite the two groups having similar LVEF, GLS was significantly lower in AS patients (*p* = 0.014). Conversely, the analysis of MW showed significantly increased values of GWI, GCW, and GWW (*p* < 0.005) in AS patients compared with controls, with no changes in terms of GWE (Table 1).

### 3.2. Staging Classification

According to the cardiac damage scheme, 36 (21.2%) AS patients were in Stage 0, 43 (25.3%) patients were in Stage 1, 65 (38.2%) patients were in Stage 2, and 26 (15.3%) patients were in Stage 3 to 4. Table 2 shows the mean values of the MW indices and GLS according to each stage.

**Figure 1:**
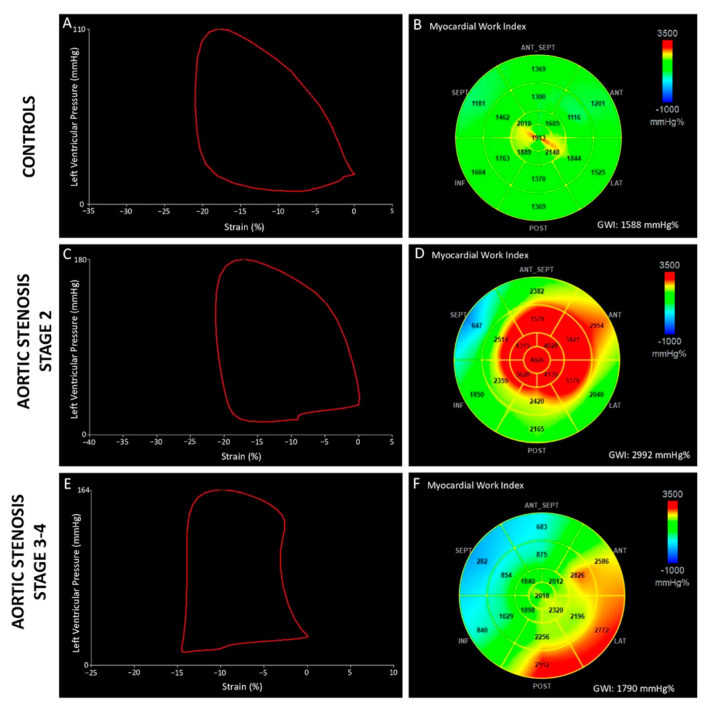
Pressure-strain loops (**left panels**) and 17-segment bull’s-eye representation of GWI (**right panels**) in a healthy subject (**A**,**B**) and in two patients with severe aortic stenosis (**C**–**F**). Compared to the control patient, aortic stenosis patient in stage 2 of cardiac damage (**C**,**D**) presented a larger pressure–strain loop, from which higher value of GWI has been estimated. Conversely, in advanced stage of aortic stenosis (**E**,**F**), GWI reduction reflected a more impaired LV contractile performance.

A significant reduction in the GWI was observed in patients in Stages 3–4 compared to Stage 0 and Stage 2 (*p* = 0.05 vs. Stage 0, *p* = 0.023 vs. Stage 2), while no difference was observed between Stage 0, Stage 1, and Stage 2 (Figure 1). In addition, GCW was reduced in Stages 3–4 as compared to other stages but without reaching significant levels (*p* = 0.119). Overall, there was no difference in GWW and GWE among all stages. A similar result was obtained for GLS, which was significantly reduced in Stages 3–4 as compared to Stage 0 (*p* = 0.002) (Table 2).

### 3.3. Prognostic Value of Global Work Index

Out of 170 asymptomatic AS patients, 161 were followed-up for a median of 30 months (interquartile range: 15–48 months). During this period, 76 (47%) patients underwent AVR (48 had surgical aortic valve replacement, 28 had transcatheter aortic valve replacement), and 27 patients (17%) died, of whom 23 (85%) died from cardiovascular cause. Moreover, 18 (11%) of the patients who died had no AVR. The baseline characteristics of patients with and without events (AVR or death) are listed in Table 3. Patients who died were older and more often diabetic had higher values of BNP, body mass index, LV mass, and LA volume and had more impaired GLS. Moreover, a significant reduction in the GWI (*p* = 0.006) and GCW (*p* = 0.002) at baseline was observed in patients who died.

On multivariable Cox-regression analysis and after adjustment for age, sex, body mass index, BNP (log_e_), LV mass index, indexed LA volume, GLS and AVR treatment, GWI, and GCW were independently associated with an increased risk of all-cause death (*p* = 0.024 and *p* = 0.003, respectively) (Table 4) and cardiovascular death (*p* = 0.034 and *p* = 0.003, respectively) (Table 5).

**Figure 2:**
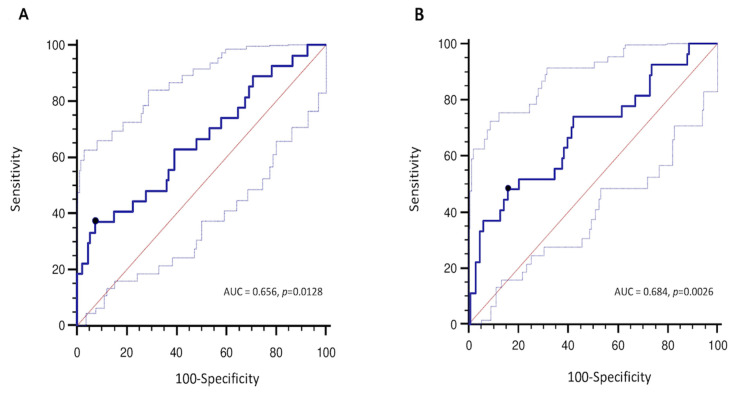
Receiver operator characteristics (ROC) curve analysis for GWI (**A**) and GCW (**B**) as a predictor of all-cause death.

The best GWI value associated with all-cause mortality was 1951 mmHg% (sensitivity 37%, specificity 92%, AUC = 0.656, *p* = 0.012), while the best GCW value associated with the outcome was 2475 mmHg% (sensitivity 48%, specificity 85%, AUC = 0.684, *p* = 0.002) (Figure 2). Despite that the prediction of all-cause of death was slightly better for GWI and GCW compared to GLS (AUC = 0.634, *p* = 0.028), AUC’s comparison did not reveal a significant superiority of both GWI and GCW over the other echocardiographic parameter, while LVEF (AUC = 0.556, *p* = 0.358) and LV mechano-energetic efficiency index (AUC = 0.568, *p* = 0.294) did not predict prognosis (Supplementary Figure S1).

**Figure 3:**
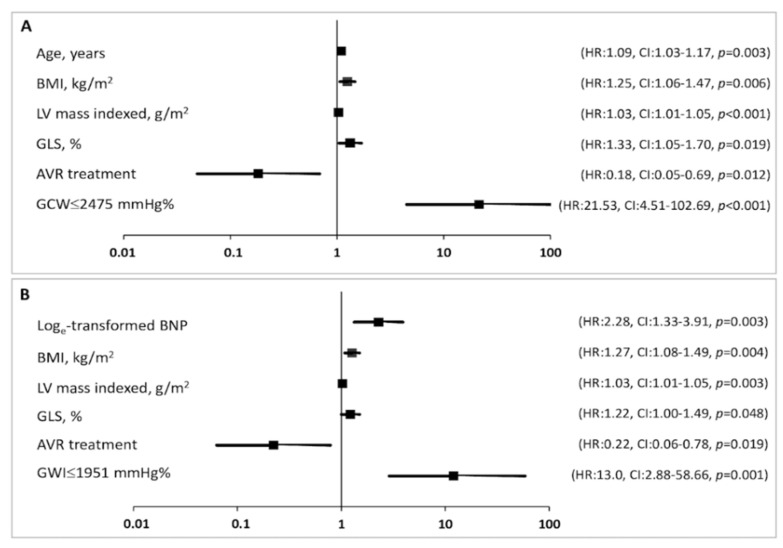
Forest plot showing the HR (bold square) and 95% CI for each variable in the final Cox multivariate model. Lower GCW (**A**) and GWI (**B**) values are associated with significantly higher risks of all-cause mortality.

However, when used as a categorical variable, both GWI ≤ 1951 mmHg% (HR 13.0, 95%CI: 2.9–58.6, *p* = 0.001) and GCW ≤ 2475 mmHg% (HR 21.5, 95%CI: 4.5–102.7, *p* < 0.001) remained significantly predictive of mortality in the multivariable analysis, even more than GLS (Figure 3). This result was confirmed when also other parameters (BMI, LV mass index, and GLS) were used as categorical variable (Supplementary Figure S2).

**Figure 4:**
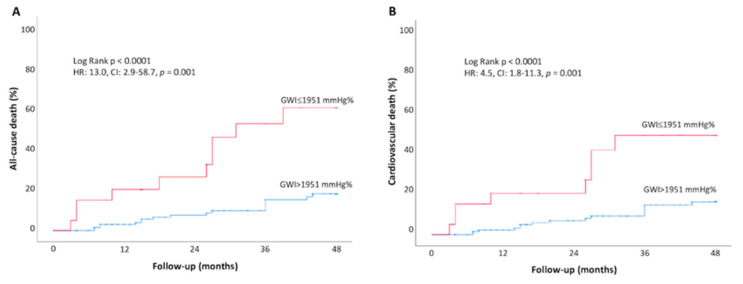
Kaplan–Meier estimates for all cause of death (**A**) and cardiovascular death (**B**) during follow-up in asymptomatic AS patients divided in two groups according to baseline more impaired GWI (≤1951 mmHg%, blue line) vs. more preserved GWI (>1951 mmHg%, red line).

**Figure 5:**
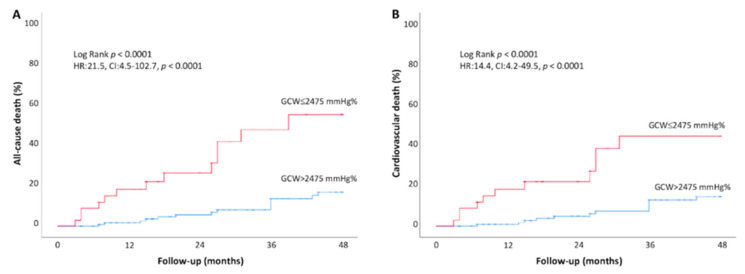
Kaplan–Meier curves of all cause (**A**) and cardiovascular death (**B**) during follow-up for asymptomatic AS patients according to baseline more impaired GCW (≤2475 mmHg%, blue line) vs. more preserved GCW (>2475 mmHg%, red line).

The cumulative event rate for all-cause and cardiovascular mortality was significantly higher in asymptomatic AS patients with GWI ≤ 1951 mmHg% at baseline, compared to those with higher GWI (>1951 mmHg%) (all-cause death: 37.0% vs. 7.5% at 48-month follow-up, respectively, log-rank *p* < 0.001; CV death: 34.8% vs. 8.7% log-rank *p* < 0.001) (Figure 4). Higher rates of all-cause and cardiovascular mortality were also observed in AS patients with lower GCW (≤2475 mmHg%) as compared to those with a more preserved GCW (>2475 mmHg%) (all-cause death:48.1% vs. 15.7% at 48-month follow-up, respectively, log-rank *p* < 0.001; CV death: 47.8% vs. 16.7% log-rank *p* < 0.001) (Figure 5).

## 4. Discussion

The main findings of this study are as follows: (i) in asymptomatic patients with moderate-to-severe AS and preserved LVEF, MW indices, including GWI, GCW, and GWW, are significantly increased as compared to controls; (ii) in advanced stages of cardiac disease, when right ventricular involvement or subclinical signs of heart failure occurred, GWI is significantly reduced as compared to patients without signs of cardiac damage; (iii) a reduction in the GWI of ≤ 1951 mmHg% or GCW ≤ 2475 mmHg% is an independent predictor of mortality.

### 4.1. Myocardial Performance in Aortic Stenosis

Traditionally, LVEF has been considered the best representative parameter of LV systolic function and, in severe AS patients, a specific cut-point of <50–55% has been used as an indication of AVR [1] because of its association with worse prognosis [14]. Despite the widespread use of LVEF, its load dependence, lack of reproducibility, and low sensibility to detect subclinical LV dysfunction [15] make the interpretation of LVEF an inadequate marker of LV contractility, especially in valvular heart disease. More recent studies in AS patients have correlated GLS with cardiac events and its worsening with subclinical impairment of LV function and the occurrence of symptoms [10,16]. However, although GLS can be a marker of early, subclinical LV dysfunction, its load dependency can affect its diagnostic accuracy, too [17]. An increase in the afterload can lead to a decrease in GLS and thus to the misinterpretation of the true contractile function of the LV.

MW has been proposed as a new approach that can offer incremental value for the evaluation of myocardial function, by considering both the myocardial deformation and the afterload, and which reflects regional and global myocardial oxygen consumption and metabolism [6,11].

The clinical application of MW measurement has been investigated in several pathological conditions [5,18,19]. To the best of our knowledge, this is the first study to evaluate the prognostic value of MW indices in patients with AS. The increase in the GWI and GCW observed in AS compared to the controls reflects the higher energy level required by the LV pump to work against the increased afterload. A similar finding was observed by Chan et al. in a subgroup of patients with advanced grades of hypertension [19]. As in patients with uncontrolled hypertension, LV remodeling in AS is accompanied with higher LV end-systolic stiffness, which enhances myocardial contractility, reflecting the ability of the LV to pump against a given pressure with higher level. Hence, a GWI increase represents an index of the enhanced myocardial contractility in a remodeled LV, characterized, in the first stage of the disease, by preserved LVEF and GLS (stage 0) and progressing to decreased GLS (stage 1).

An increase in GWW was detected in the AS cohort, perhaps ascribable to the enhanced wall stress produced in an attempt to overcome the increased afterload. Given that GWE derives from the ratio of GCW and GWW, the proportional increase in both constructive and wasted work could explain why GWE remained preserved in the AS group, with no difference compared to control group. This result also appears to be concordant with the data in hypertensive patients [19].

### 4.2. Myocardial Work and Outcomes

Currently, the main triggers for intervention in patients with AS and preserved LVEF are the presence of AS-related symptoms and the severity of AS [1], mainly assessed by Doppler echocardiographic data [7]. However, current evidence on increased mortality in moderate AS patients raised some concerns on the strict classification of the disease based only on valve haemodynamics, suggesting that a more complete evaluation, also integrating markers of cardiac injury or metabolic distress could promptly detect patients that would benefit from of an early AVR intervention [2,20]. The staging cardiac scheme, which includes myocardial structural changes, haemodynamics parameters, and indices of myocardial dysfunction (including GLS), has demonstrated a strong predictive value in asymptomatic moderate-to-severe AS patients [4]. Tastet el al. reported an increased risk of death when cardiac damage stage was ≥ 2, with an excess of mortality in Stages 3 to 4 [4]. In the present study, using the same staging scheme, we found a significant reduction in the GWI and a non-significant reduction in GCW in advanced stages of AS, which are the expressions of a more impaired contractile performance of the cardiomyocytes. Indeed, after an initial adaptive LV response to chronic pressure overload, the ongoing development of concentric hypertrophy increases LV stiffness and eventually leads to the formation of myocardial fibrosis, which negatively impacts LV function, resulting in metabolic impairment. Moreover, as shown in previous studies, the estimation of MW by pressure–strain loops (PSL) is an index of regional and global myocardial oxygen consumption and metabolism [11]. An impairment of the GWI could, then, be considered an early index of cardiac failure. Fortuni et al. recently described an independent association of lower values of GWI and GCW in patients with NYHA III–IV heart failure symptoms, suggesting that MW indices might be more sensitive for the detection of LV remodeling and maladaptation in AS [13].

In our study, both GWI and GCW were predictive of all-cause and cardiovascular mortality. These results likely highlighted the fact that GCW estimation represents the assessment of LV function during the systolic and isovolumic relaxation phase. This might be of particular interest in AS patients, in whom a concurrent impairment of LV deformation and diastolic function is found. We furthermore demonstrated that a reduction in the GWI below 1951 mmHg% or GCW below 2475 mmHg% was predictive of all-cause and cardiovascular mortality, even when adjusted for cardiovascular risk factors, echocardiography parameters, and AVR. These results suggest that even when LVEF is preserved, subclinical impairment of myocardial contractility may impact on patient’s prognosis. In our study, the risk of cardiovascular and all-cause death in patients with a GWI < 1951 mmHg% or with a GCW < 2475 mmHg% was significantly higher than in patients with GLS >−15%. Of course, this result, which seems very impressive, should be confirmed by further, larger studies. One of the possible advantages of MW indices over GLS is that, by taking into consideration both the myocardial deformation and the afterload, it should be less load-dependent than GLS and thus more reliable in describing myocardial performance and in the prediction of worse prognosis.

The evaluation of myocardial work is still in its infancy, but it may provide additional information on myocardial performance, useful in the risk assessment and in the determination of the optimal timing for AVR.

### 4.3. Limitations

Although the clinical data were prospectively collected, our study is observational and retrospective; therefore, it might be prone to bias from uncontrolled confounders. Another limitation is the estimation of LV systolic pressure based on the sum of brachial cuff pressure and mean transaortic gradient. However, previous studies in AS patients have demonstrated an excellent correlation between MW indices calculated with the invasive and echocardiographic-derived LV systolic pressure [12,13]. Long-term follow-up was not available for all patients. However, as mortality curves diverge already at 12 months, the data can be considered reliable. Finally, the GWI and GCW cut-off yielded in the study are characterized by high specificity but very low sensitivity for detecting patients at risk of death. A possible explanation is that as MW indices decrease in more advanced stages of cardiac damage, values lower than the cutoff reflect a more advanced disease with a more impaired contractile performance of the cardiomyocytes. These values would thus identify with greater certainty those at the highest risk of long-term cardiovascular and all cause death that, probably, would benefit from an early intervention, regardless symptomatic status.

## 5. Conclusions

In asymptomatic patients with moderate to severe AS, LV remodelling and increased wall stress is associated with increased MW indices. Advanced stages of cardiac damage are characterized by reduced values of GWI and GCW, which are associated with increased mortality. Therefore, the evaluation of MW indices may allow for a better identification of asymptomatic patients at increased risk of cardiovascular and all cause death. Larger studies are needed to validate the proposed method for the non-invasive estimation of MW in AS patients and to better define the role of MW parameters in predicting the progression and the prognosis of aortic valve disease.

## Figures and Tables

**Table 1:** Baseline clinical and echocardiographic characteristics.

Variables	Controls (*n* = 50)	Asymptomatic AS Group (*n* = 170)	*p* Value
Clinical variables			
Age ± SD, years	71.1 ± 4.7	69.3 ± 13.4	0.358
Male, gender *n* (%)	25 (50)	102 (60)	0.135
BMI ± SD, kg/m^2^	25.5 ± 3.4	26.5 ± 4.2	0.135
BSA ± SD, m^2^	1.7 ± 0.1	1.8 ± 0.2	0.075
Systolic arterial pressure ± SD, mmHg	128 ± 11	136 ± 18	0.013
Diastolic arterial pressure ± SD, mmHg	77 ± 8	73 ± 10	0.004
Log_e_-transformed BNP ± SD	-	4.3 ± 1.3	-
Diabetes mellitus, *n* (%)	-	39 (23)	-
Hypertension, *n* (%)	-	112 (66)	-
Hypercholesterolemia, *n* (%)	-	106 (62)	-
Current smoking, *n* (%)	-	29 (17)	-
Coronary artery disease, *n* (%)	-	19 (11)	-
Previous PCI, *n* (%)	-	15 (9)	-
Chronic kidney disease, *n* (%)	-	40 (23)	
Atrial fibrillation	-	6 (4)	-
LV dimensions and geometry			
Interventricular septum ± SD, mm	9.6 ± 1.2	12.4 ± 2.0	<0.001
LV posterior wall ± SD, mm	9.8 ± 2.0	10.6 ± 1.6	0.004
LV end-diastolic diameter ± SD, mm	42.7 ± 5.3	45.4 ± 5.9	0.005
LV end-systolic diameter ± SD, mm	29.3 ± 5.1	29.9 ± 5.8	0.535
LV mass indexed ± SD, g/m^2^	76.8 ± 20.2	103.9 ± 26.4	<0.001
Relative wall thickness ± SD	0.46 ± 0.1	0.47 ± 0.09	0.514
Aortic valve severity			
Mean Pressure Gradient ± SD, mmHg	-	37.3 ± 14.6	-
Peak aortic Velocity ± SD, m/s	-	3.8 ± 0.7	-
Aortic Valve Area ± SD, cm^2^	-	1.02 ± 0.35	-
Indexed aortic valve area ± SD, cm^2^/m^2^	-	0.55 ± 0.18	-
Indexed Stroke Volume ± SD, mL/m^2^	-	48.0 ± 11.3	-
Zva ± SD, mmHg/mL/m^2^	-	3.8 ± 1.0	-
LV systolic and diastolic function			
LV end-diastolic volume ± SD, mL	83.0 ± 24.3	93.5 ± 34.2	0.065
LV end-systolic volume ± SD, mL	30.2 ± 10.4	36.3 ± 16.7	0.025
LV EF ± SD, %	64 ± 5	62 ± 6	0.130
GLS ± SD, %	−20.7 ± 2.1	−18.7 ± 2.8	0.014
Indexed LA volume ± SD, mL/m^2^	26.5 ± 8.6	34.8 ± 13.0	<0.001
Mitral E/A ratio ± SD	0.9 ± 0.2	0.9 ± 0.3	0.740
E/e’ ± SD, average	7.4 ± 1.8	11.9 ± 4.4	<0.001
TTPG ± SD, mmHg	17.0 ± 8.9	26.3 ± 8.3	<0.001
TAPSE ± SD, mm	22.8 ± 3.2	22.5 ± 3.9	0.601
S’ ± SD, cm/s	13.6 ± 3.0	12.6 ± 2.8	0.048
Right atrial volume ± SD, mL	32.7 ± 10.8	38.7 ± 16.4	0.017
Myocardial work			
GWI ± SD, mmHg%	2005 ± 302	2528 ± 521	<0.001
GCW ± SD, mmHg%	2360 ± 353	2948 ± 598	<0.001
GWW ± SD, mmHg%	90 ± 49	139± 110	0.005
GWE ± SD, %	96 ± 2	95 ± 4	0.171

Values are *n* (%) or mean ± SD. BMI = body mass index; BSA = body surface area; BNP = brain natriuretic peptide; EF = ejection fraction; GLS = global longitudinal strain; GWI = global work index; GCW = global constructive work; GWW = global wasted work; GWE = global work efficiency; LA = left atrial; LV = left ventricle; PCI = percutaneous coronary intervention; SD = standard deviation; TTPG = trans-tricuspid pressure gradient; Zva = Valvulo-arterial impedance.

**Table 2:** MW parameters and GLS in AS patients according to stages of cardiac damage.

Variables	Stage 0 (*n* = 36)	Stage 1 (*n* = 43)	Stage 2 (*n* = 65)	Stage 3–4 (*n* = 26)	*p* Value
GWI ± SD, mmHg%	2609 ± 503	2495 ± 549	2611 ± 510	2264 ± 457 *^,#^	0.024
GCW ± SD, mmHg%	2999 ± 633	2938 ± 645	3026 ± 564	2700 ± 507	0.119
GWW ± SD, mmHg%	131 ± 166	149 ± 92	139 ± 90	133 ± 90	0.901
GWE ± SD, %	95 ± 7	94 ± 3	95 ± 3	94 ± 3	0.917
GLS ± SD, %	−20.0 ± 2.5	−18.5 ± 2.6	−18.6 ± 2.7	−17.4 ± 3.2 *	0.004

* *p* < 0.05 vs. Stage 0, ^#^ *p* < 0.05 vs. preceding value. Abbreviations as in Table 1.

**Table 3:** Comparison between asymptomatic AS patients who died or not during follow-up.

Variables	Survivors (*n* = 134)	All-Cause Deaths (*n* = 27)	*p* Value
Clinical variables			
Age ± SD, years	68.4 ± 13.3	76.0 ± 9.4	0.005
Male, gender *n* (%)	77 (57)	17 (63)	0.597
BMI ± SD, kg/m^2^	26.1 ± 4.0	28.0 ± 5.1	0.037
BSA ± SD, m^2^	1.8 ± 0.2	1.8 ± 0.2	0.907
Systolic arterial pressure ± SD, mmHg	136.0 ± 18	135 ± 22	0.735
Diastolic arterial pressure ± SD, mmHg	73 ± 10	71 ± 9	0.262
Log_e_-transformed BNP ± SD	4.2 ± 1.3	5.1 ± 1.3	0.002
Diabetes mellitus, *n* (%)	27 (20)	10 (37)	0.045
Hypertension, *n* (%)	92 (69)	17 (63)	0.907
Hypercholesterolemia, *n* (%)	87 (133)	16 (60)	0.705
Current smoking, *n* (%)	26 (19)	3 (11)	0.319
Coronary artery disease, *n* (%)	15 (11)	4 (15)	0.502
Previous PCI, *n* (%)	12 (9)	3 (11)	0.639
Chronic kidney disease, *n* (%)	29 (22)	9 (33)	0.368
Atrial fibrillation, *n* (%)	5 (4)	2 (7)	0.391
LV dimensions and geometry			
Interventricular septum ± SD, mm	10.5 ± 1.6	13.1 ± 1.9	0.063
LV posterior wall ± SD, mm	9.8 ± 2.0	11.0 ± 1.1	0.118
LV end-diastolic diameter ± SD, mm	45.1 ± 5.5	47.0 ± 7.1	0.132
LV end-systolic diameter ± SD, mm	29.9 ± 5.7	30.6 ± 6.1	0.619
LV mass indexed ± SD, g/m^2^	101.7 ± 25.5	117.4 ± 28.1	0.007
Relative wall thickness ± SD	0.47 ± 0.10	0.48 ± 0.09	0.714
Aortic valve severity			
Mean Pressure Gradient ± SD, mmHg	38.6 ± 14.8	38.3 ± 17.1	0.942
Peak aortic Velocity ± SD, m/s	3.9 ± 0.7	3.8 ± 0.7	0.605
Aortic Valve Area ± SD, cm^2^	1.00 ± 0.35	1.01 ± 0.26	0.725
Indexed aortic valve area ± SD, cm^2^/m^2^	0.54 ± 0.18	0.55 ± 0.15	0.664
Indexed Stroke Volume ± SD, mL/m^2^	47.8 ± 10.6	48.9 ± 14.3	0.664
Zva ± SD, mmHg/mL/m^2^	3.8 ± 1.0	3.8 ± 1.3	0.930
LV systolic and diastolic function			
LV end-diastolic volume ± SD, mL	93.8 ± 33.6	93.1 ± 39.3	0.925
LV end-systolic volume ± SD, mL	36.2 ± 16.4	37.3 ± 20.2	0.763
LV EF ± SD, %	62 ± 6	61 ± 7	0.281
GLS ± SD, %	−19.0 ± 2.7	−17.5 ± 3.3	0.016
Indexed LA volume ± SD, mL/m^2^	33.4 ± 11.9	42.6 ± 16.9	0.002
Mitral E/A ratio ± SD	0.9 ± 0.3	0.8 ± 0.2	0.050
E/e’ ± SD, average	11.8 ± 4.3	11.9 ± 3.7	0.924
TTPG ± SD, mmHg	26.5 ± 8.2	26.8 ± 8.8	0.900
TAPSE ± SD, mm	22.8 ± 3.28	21.3 ± 4.0	0.072
S’ ± SD, cm/s	12.6 ± 2.7	12.9 ± 3.3	0.694
Right atrial volume ± SD, mL	38.2 ± 16.3	39.8 ± 17.4	0.681
Myocardial work			
GWI ± SD, mmHg%	2603 ± 503	2307 ± 532	0.006
GCW ± SD, mmHg%	3040 ± 582	2647 ± 602	0.002
GWW ± SD, mmHg%	137 ± 113	152 ± 96	0.513
GWE ± SD, %	95 ± 4	94 ± 3	0.199

Values are *n* (%) or mean ± SD. Abbreviations as in Table 1.

**Table 4:** Univariable and multivariable Cox proportional hazard model for all-cause mortality for asymptomatic aortic stenosis patients.

Variable	Univariable		Multivariable	
	HR (95%CI)	*p*-Value	HR (95%CI)	*p*-Value
**Model 1**				
Age, years	1.063 (1.021–1.107)	0.003	1.051 (0.991–1.115)	0.099
Log_e_-transformed BNP	2.180 (1.448–3.281)	<0.001	1.877 (1.011–3.484)	0.046
BMI, kg/m^2^	1.091 (1.001–1.188)	0.047	1.231 (1.046–1.449)	0.012
LV mass indexed, g/m^2^	1.023 (1.009–1.038)	0.001	1.027 (1.007–1.047)	0.008
Indexed LA volume, mL/m^2^	1.053 (1.023–1.084)	<0.001	0.986 (0.937–1.039)	0.602
AVR treatment	3.083 (1.342–7.080)	0.008	0.336 (0.093–1.212)	0.096
GLS, %	1.202 (1.045–1.382)	0.010	1.286 (1.014–1.631)	0.038
GWI, mmHg%	0.999 (0.998–1.000)	0.003	0.998 (0.997–0.999)	0.024
**Model 2**				
Age	1.063 (1.021–1.107)	0.003	1.070 (1.004–1.141)	0.037
Log_e_ -transformed BNP	2.180 (1.448–3.281)	<0.001	1.772 (0.939–3.344)	0.077
BMI, kg/m^2^	1.091 (1.001–1.188)	0.047	1.232 (1.039–1.439)	0.026
LV mass indexed, g/m^2^	1.023 (1.009–1.038)	0.001	1.032 (1.009–1.056)	0.003
Indexed LA volume, mL/m^2^	1.053 (1.023–1.084)	<0.001	1.010 (0.965–1.057)	0.672
AVR treatment	3.083 (1.342–7.080)	0.008	0.287 (0.080–1.025)	0.055
GLS, %	1.202 (1.045–1.382)	0.010	1.305 (1.035–1.645)	0.025
GCW, mmHg%	0.999 (0.998–1.000)	<0.001	0.998 (0.997–0.999)	0.003

For continuous variables, Hazard Ratio (HR) is shown for an increase in 1U. Abbreviations as in Table 1.

**Table 5:** Univariable and multivariable Cox proportional hazard model for cardiovascular mortality for asymptomatic aortic stenosis patients.

Variable	Univariable		Multivariable	
	HR (95%CI)	*p*-Value	HR (95%CI)	*p*-Value
**Model 1**				
Age, years	1.077 (1.028–1.128)	0.002	1.051 (0.985–1.122)	0.132
Log_e_-transformed BNP	2.099 (1.371–3.213)	0.001	2.245 (1.236–4.077)	0.008
BMI, kg/m^2^	1.088 (0.992–1.192)	0.074	1.157 (0.998–1.340)	0.053
LV mass indexed, g/m^2^	1.027 (1.012–1.043)	0.001	1.027 (1.005–1.049)	0.014
Indexed LA volume, mL/m^2^	1.053 (1.020–1.086)	0.001	1.019 (0.973–1.067)	0.421
AVR treatment	3.619 (1.419–9.228)	0.007	0.451 (0.120–1.698)	0.239
GLS, %	1.157 (0.996–1.343)	0.057	1.291 (1.010–1.649)	0.042
GWI, mmHg%	0.999 (0.998–1.000)	0.018	0.998 (0.997–1.000)	0.034
**Model 2**				
Age, years	1.077 (1.028–1.128)	0.002	1.075 (1.005–1.151)	0.035
Log_e_-transformed BNP	2.099 (1.371–3.213)	0.001	1.877 (0.931–3.782)	0.078
BMI, kg/m^2^	1.088 (0.992–1.192)	0.074	1.125 (0.970–1.304)	0.120
LV mass indexed, g/m^2^	1.027 (1.012–1.043)	0.001	1.030 (1.009–1.051)	0.005
Indexed LA volume, mL/m^2^	1.053 (1.020–1.086)	0.001	1.014 (0.967–1.057)	0.578
AVR treatment	3.619 (1.419–9.228)	0.007	0.402 (0.108–1.500)	0.175
GLS, %	1.157 (0.996–1.343)	0.057	1.477 (1.122–1.945)	0.005
GCW, mmHg%	0.999 (0.998–1.000)	0.003	0.998 (0.997–0.999)	0.003

For continuous variables, Hazard Ratio (HR) is shown for an increase in 1U. Abbreviations as in Table 1.

## Data Availability

The data presented in this study are available on request from the corresponding author. The data are not publicly available due to firewalls to access our dataset from outside our Institution.

## Supplementary Materials

The following supporting information can be downloaded at: https://www.mdpi.com/article/10.3390/jcm11061555/s1. Figure S1: Comparison of ROC curves for echocardiographic parameters; Figure S2: Multivariate Cox regression analysis for prediction of all-cause death.
