# Supplementary material for: Prognostic Value of Non-Invasive Global Myocardial Work in Asymptomatic Aortic Stenosis
Source: J Clin Med. 2022 Mar 11;11(6):1555. doi: 10.3390/jcm11061555 (PMC8953091; doi:10.3390/jcm11061555)
Supplement: Supplementary file 1 [file jcm-11-01555-s001.zip › jcm-1573256-supplementary.pdf]

Supplementary Figure S1

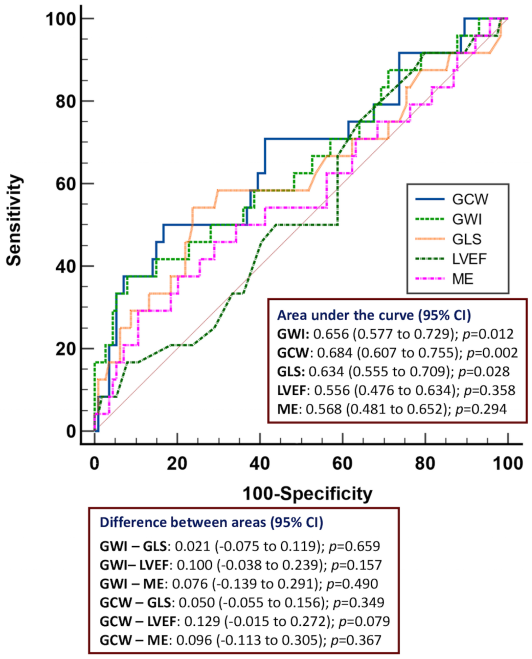

Receiver operator characteristics (ROC) curves for global work index (GWI), global constructive work (GCW), global longitudinal strain (GLS), left ventricular ejection fraction (LVEF) and mechano-energetic efficiency (ME). CI = confidence interval

Supplementary Figure S2

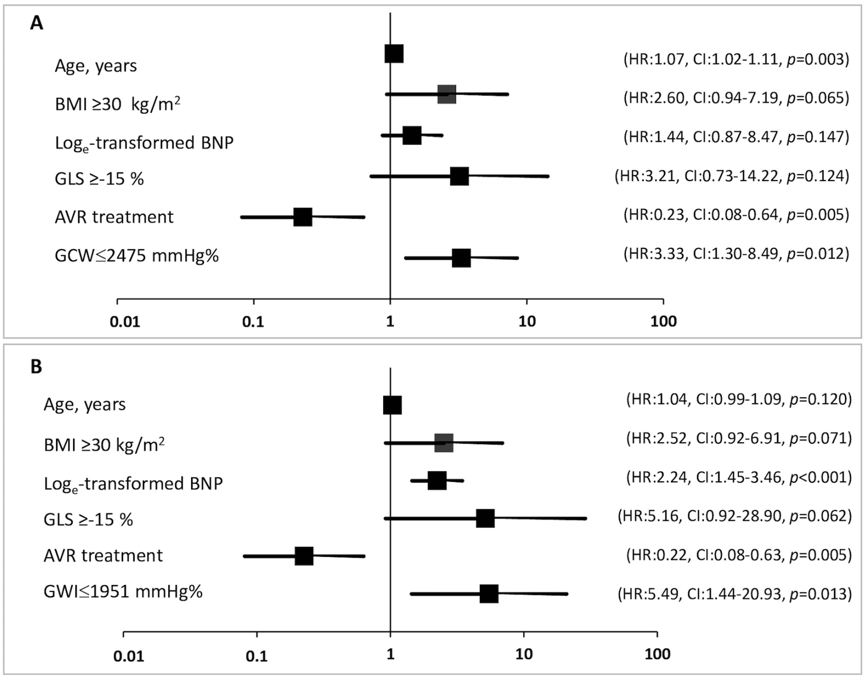

Forest plot showing the HR (bold square) and 95% Confidence Intervals (CI) for each variable in the final Cox multivariate model. Lower GCW (**A**) and GWI (**B**) values are associated with significantly higher risks of all-cause mortality.
